# Wear and Fragmentation Resistance of Mineral Aggregates—A Review of Micro-Deval and Los Angeles Tests

**DOI:** 10.3390/ma14185456

**Published:** 2021-09-21

**Authors:** Paweł Strzałkowski, Urszula Kaźmierczak

**Affiliations:** Department of Mining, Faculty of Geoengineering, Mining and Geology, Wroclaw University of Science and Technology, Wybrzeże Wyspiańskiego 27, 50-365 Wrocław, Poland; urszula.kazmierczak@pwr.edu.pl

**Keywords:** resistance to wear, resistance to fragmentation, Los Angeles test, micro-Deval test, mineral aggregates, aggregate properties

## Abstract

The aim of this article is to present the current knowledge and experiences related to wear and fragmentation resistance tests and to indicate those of their aspects that require further research. For this purpose, a review of the literature was performed. Results show that tests of resistance to wear (the M_DE/DS_ test) and fragmentation (the LA test) are performed worldwide according to different standards (and thus following different test methods), which prevents a comparison of the obtained results. Comparative research into the M_DE/DS_ and LA tests indicates that the M_DE/DS_ test is more effective. The disadvantage of both tests lies in the dimension range of the aggregate. In addition, the use of steel balls in the LA test may not reflect the actual influence that the internal properties of the material have on the fragmentation process. A final review of the available knowledge allowed the formulation of proposals regarding further research directions, such as proposed changes of test methods, extensive analysis, and selection of optimal dimensions for tested aggregates, analysis of short-term and long-term tests, as well as extensive research into and an analysis of the impact of crushing on the physical, mechanical, and geometric properties of aggregates.

## 1. Introduction

Mineral aggregates are among the most important raw materials mined worldwide and also among the materials most frequently used in civil engineering [1,2,3,4,5]. Adomako et al. [6] observe that crushed stones account for nearly 50% of aggregate production in Europe. Although the production of granular rock materials is at such a high level, it still grows, and the materials are used in various areas of industry. Being a basic construction material, mineral aggregates must have certain, defined technical parameters. The most important of these parameters include their physical, mechanical, and geometric properties. However, rock raw materials extracted in mines and processed into aggregates do not have uniform properties. Their properties vary depending on the part of the deposit and are strictly related to the geological structure. Therefore, an optimal solution is to manage the production process in such a manner that the obtained aggregates are of high quality while the deposit is maximally. However, the quality of an aggregate should be understood as the degree to which it fulfills the requirements of mineral aggregates, depending on their application. Of course, this quality is variable and depends on the structure and composition of rocks, but it also depends on the technology adopted for aggregate production. This means that the quality of an aggregate should be understood as the degree to which the parameters of a manufactured mineral aggregate are fulfilled (including roughness, angularity, grain size, wear, and fragmentation resistance). Räisänen and Torppa [7] observe that if a heterogeneous rock is selectively quarried on the basis of a comprehensive quality assessment, aggregate resources may be maximized by not using high-quality aggregates in applications in which low-quality aggregates are sufficient. With such an operational approach, the local competitiveness of an aggregate-producing company may be increased. This solution also allows more rational deposit extraction and management.

Mineral aggregates are prone to external loads and difficult environmental conditions during each of the processes, starting at the production stage, through the transportation stage, and also during the application phase performed at the construction site. Aggregates having a low resistance to degradation may be the cause of a number of problems, for example, in the case of Portland cement mixtures, hot mineral–asphalt mixtures [8], or when used as ballast in the construction of roads or railway tracks. Therefore, much importance should be paid to tests of the physical, mechanical, and geometric properties of the rock material, as well as the quality of the aggregates produced from this material.

The required high quality of aggregates and their wide application potential cause the quality of granular rock materials to be given much attention in the literature [6,8,9,10,11,12,13,14,15,16,17]. This discussion focuses on a number of aspects, such as test methodologies for the properties of aggregates, rock properties, aggregate quality, or the application potential. Despite a very broad range of research, the knowledge and experiences in the field have not yet been given a comprehensive, systematic, and multi-aspect approach, which is the objective of this article. Such a systematic approach is hoped to allow both the identification of factors influencing the quality of mineral aggregates and the indication of the possible and the required directions for further research on mineral aggregates. The methodology includes a review of the literature on the resistance of mineral aggregates to wear and fragmentation. A significant number of previous research works addressed this problem, albeit not in a complex manner. This publication is an attempt at compiling the available information on the resistance of mineral aggregates to wear and fragmentation and at determining the characteristics of, the relationships involved in, and the reasons behind the destruction of this material during the micro-Deval and the Los Angeles tests.

## 2. Methodology

The literature review was performed following the SLR (Systematic Literature Review) method. The literature (sources) was critically analyzed on the basis of defined criteria. This method offers a perspective on what is known, what already exists, and what is included in the literature on a particular issue. The aim of this method is to demonstrate to what extent the addressed problem is different from the existing state of knowledge on the subject.

The literature review was performed in the Scopus database. Three general keywords were defined: “micro-Deval AND aggregate AND resistance to wear”, “Los Angeles AND aggregate AND resistance to fragmentation”, and “aggregate AND micro-Deval AND Los Angeles.” The analysis covered a period between 1990 and Feb. 1, 2021. The search provided a total number of 338 records. However, some of them included two or three repetitions (Table 1). Of all the results, only those related to mineral aggregates were included for further analysis.

The analysis demonstrated an exponentially increasing interest in the topic of aggregate destruction due to wear and fragmentation. This fact may indicate a lack of clear descriptions of the reasons behind aggregate destruction, as well as the importance of identifying wear and fragmentation resistance when defining the properties of rock material.

## 3. Wear and Fragmentation Resistance of Mineral Aggregates—A Review of Micro-Deval and Los Angeles Tests

Mineral aggregates are basic materials used in construction, and as such, they are subject to various physical and chemical phenomena, depending on the location in which they are used. For this reason, they should have high hardness and durability. In addition, they should be uniform, clean, and highly resistant to wear and fragmentation [12,18,19]. Knowledge of their mechanical properties allows predictions of the behavior of aggregates under different loads. Aggregates are subjected to significant degradation during their entire lifetime and, according to Teymen [19], Koohmishi [20], and Fladvad and Ulvik [21], this is particularly important if they are used as ballast in railroads, as basecourse in roads, or as a component of concrete. Additionally, knowing the physical and mechanical parameters of aggregates allows evaluations of the strength of concrete mixtures or bituminous asphalt mixtures [8,10,15,22].

Another issue addressed in the literature is the problem of depleting natural resources of rock raw materials. It is the reason for researching new materials that can be used as a substitute. The review indicated a growing interest in aggregates obtained from recycling processes. A significant number of publications suggest the possibility of replacing mineral aggregates with recycled aggregates and offer evaluations of the physical and strength-related parameters of such materials [1,23,24,25,26,27,28]. The results demonstrate that such materials may become an alternative construction material. From a social and environmental perspective, the use of recycled aggregates is a good solution. However, broader analyses seem necessary to determine how the properties of these products determine their application directions. Additionally, it should be emphasized that recycled aggregates which are used as additional material to satisfy the demand for aggregates generally have worse physical and mechanical properties in comparison with mineral aggregates.

A review of the analyses or modifications of research methods using the micro-Deval apparatus and the Los Angeles mill, which serve to identify resistances to wear (the M_DE/DS_ method) and to fragmentation (the LA method), respectively, indicates that the test methodologies vary significantly [4,29,30]. The review also points to the analyses of the influence of crushing processes on various aggregate properties, including strength parameters [31,32,33,34,35,36,37,38]. The review results also suggest that the mineral composition, structure, and size of mineral components definitely have a significant influence on the resistance to wear and fragmentation. Interestingly, the majority of publications related to research into aggregate wear and fragmentation resistance tests address issues of mutual relationships and correlations between aggregate properties [3,11,14,39,40,41,42,43,44,45,46,47,48,49,50,51,52].

### 3.1. Analysis and Modifications of Test Methods

**Figure 1:**
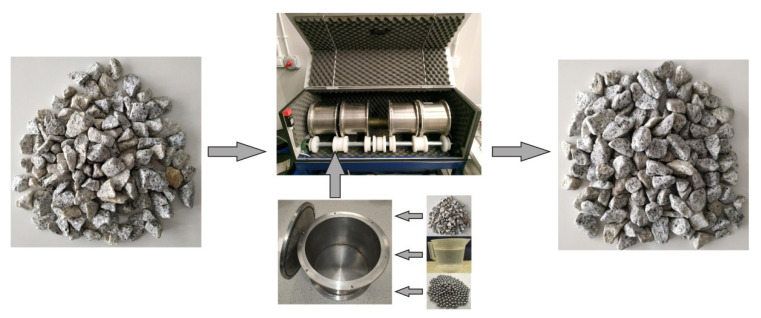
The M_DE_ test procedure.

Generally, a wear resistance test is performed on the micro-Deval apparatus (the M_DE/DS_ test) under both wet (M_DE_) and dry (M_DS_) conditions (Figure 1). In accordance with EN 1097-1 [53], the test consists of placing 500 ± 2 g of clean and dry aggregate in the drum of the micro-Deval apparatus and an appropriate amount of abrasive material (depending on the grain size of the tested aggregate). In the case of wet conditions, the drum should contain an additional 2.5 ± 0.05 L of water. The drum, hermetically closed, is subjected to 12,000 ± 10 revolutions at a rate of 100 ± 5 rpm. In the next step, the abrasive material is separated from the aggregate, and the aggregate is screened on a sieve with 1.6 mm mesh size, washed, and dewatered until dry mass. The last step consists of the calculation of the M_DE_ or M_DS_ coefficient, which describes the wear resistance according to the following equation:

(1) $$M_{DE/DS}=\frac{500-m}{5}$$

where m—mass of dry aggregate remaining on the 1.6 mm sieve.

**Figure 2:**
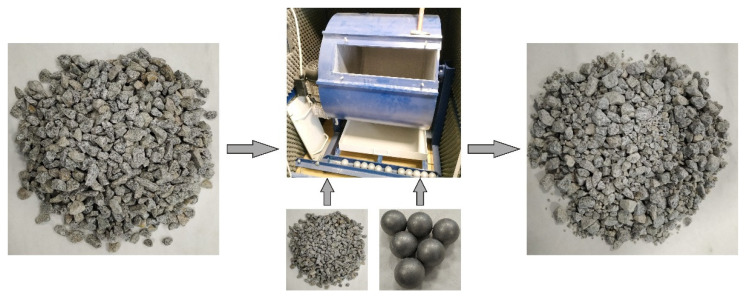
The LA test procedure.

In accordance with European standard EN 1097-2 [54], the fragmentation resistance test is performed in the Los Angeles drum (the LA test) (Figure 2). It consists of placing 500 ± 2 g of clean and dry aggregate together with steel balls in the drum (the number of the balls depends on the grain size of the tested aggregate). The drum is closed and subjected to 500 rotations at a constant speed between 31 and 33 rpm. The drum is subsequently emptied, and the aggregate is separated from the steel balls. In the next step, the aggregate is screened on a sieve with a 1.6 mm mesh size. The material remaining on the sieve is washed and dewatered until dry mass. Fragmentation resistance is expressed with an LA coefficient, which is calculated from the following equation:

(2) $$LA=\frac{5000-m}{50}$$

where m—mass of dry aggregate remaining on the 1.6 mm sieve.

The above test methods of wear and fragmentation resistance are standard and effective across Europe. Importantly, the micro-Deval and Los Angeles tests of wear and fragmentation resistance are also performed in other parts of the world. They may follow such standards as ASTM D6928 [55], AASHTO T327 [56], ASTM C131/C131M [57], and AASHTO T-96 [58], which vary from the European standards (Table 2). Gökalp et al. [29] performed a comparative analysis of the methods described in EN 1097-1 and in ASTM D6928, which are related to wear resistance tests in the micro-Deval drum (the MDE/DS test). On the other hand, the fragmentation resistance test methods (EN 1097-2 and ASTM C131) were compared in Gökalp and Uz [4]. Both the differences identified in the methodologies and the laboratory tests allowed the authors of the above publications to conclude that the two test methods yield different results depending on both the type and the grain size of the tested material.

The determinations of wear and fragmentation resistance follow similar aggregate preparation and testing procedures. However, a closer investigation of the two methods indicates that each of them illustrates different processes of rock material degradation due to mechanical interactions. The M_DE/DS_ test illustrates only the resistance to wear of the external layer of the aggregate grains due to the steel balls. On the other hand, the LA test illustrates the resistance of aggregate to degradation resulting both from wear due to the rock–rock interactions and from the impacts and crushing action due to the steel balls [8,59]. When comparing the two methods, Gökalp et al. [29] concluded that the wear resistance test (the M_DE/DS_ test) is more effective than the fragmentation resistance test (the LA test), as the test conditions, in this case, more realistically resemble the actual conditions. This is related to the fact that the wet conditions in the M_DE/DS_ test are thought to better simulate the field conditions of aggregates than the dry state in the LA test.

Umar et al. [18] developed a modified method for testing fragmentation resistance of mineral aggregates, which shortens the test duration time. The duration time is shortened by 24 h at the stage of drying the aggregate (prior to the test and after the aggregate sample is passed through the 1.6 mm sieve). However, the researchers stress that the modified LA method was developed to meet the climatic conditions of Oman. Therefore, a possibility exists that the method and the described mathematical relationship (between the standard method and the modified LA method) may not find application in a region in which the climatic conditions are different. Another modification of the LA fragmentation test method was proposed by Nataadmadja et al. [60]. The researchers suggested testing wet aggregates and compared the obtained results with the results of the original LA test. Based on the results of this comparison, a conclusion was made that the LA test can be modified in order to detect the susceptibility of aggregates to moisture. Different aggregates show different levels of water absorption, and this fact influences the final result (the percentage loss of rock material due to the LA test).

Fladvad and Ulvik [21] notice a disadvantage of using limited dimension ranges of the tested aggregate. The wear and fragmentation test method does not allow aggregate tests in wider grain size ranges. Therefore, the tests have limited functionality in identifying various aggregate variants during the design process. In addition, Rembiś [61], as well as Rangaraju and Edlinski [62], observed that aggregates display different mechanical properties depending on their fraction size. This fact may be related to the properties of the mined material, which depend on its mineral composition and its crushing method. Erichsen et al. [63] also confirm that the grain size has an influence on the obtained M_DE_ and LA coefficients. Aggregates having a larger grain size (of 31.5–50 mm) have a significantly varying grain size distribution depending on the mechanical test method. Whether a material having a greater grain size is subjected to an identical degradation process, as in the case of a smaller grain size (of 10–14 mm), therefore remains uncertain. In resistance tests, a material having a smaller grain size seems prone to both wear and, partially, fragmentation. Further disadvantages of the LA test are mentioned by Li et al. [64]. They observe that the test mechanism, which includes the degradation of the steel balls from the impacts or the crushing action of the aggregate, does not properly simulate the actual compaction or field loading conditions and, as a result, the fragmentation test method may not accurately reflect how internal properties of the material influence its fragmentation characteristics. For this reason, the researchers offer their own test method: a Gyratory Abrasion and Image Analysis (GAIA). Yet, another replacement to the traditional LA test is proposed in Mohajerani et al. [65]. It consists of testing compacted rock material which simulates the resistance to abrasion of unbound granular materials.

During strength tests, mineral aggregates are rotated for a defined number of rotations. Long-term durability assessment requires additional tests which are beyond the test methods listed in the relevant standards. Czinder et al. [66] and Wu et al. [13,30] subjected aggregates to a long-term wear process (the micro-Deval test), which was intended to better reflect the behavior of the material over a longer period of time. They demonstrated that aggregate wear represented as a function of the number of revolutions has an exponential character. Czinder et al. [66]) used this observation to define a new wear-related parameter describing long-term aggregate durability. Additionally, Qian et al. [67,68] emphasize that the reason behind the non-linear wear variability trend lies in the fact that an increasing number of wear cycles smoothens the angularity and texture of the aggregate, gradually decreasing the aggregate-to-aggregate abrasion effect and, thus, the abrasion rate. Interestingly, in its initial phase, the wear process follows a quadratic function [69], and a short-term test, in which the number of rotations is reduced in comparison to that defined in the standard, indicates a linear form [70]. Dias Filho et al. [71] demonstrated that the aggregate wear process, as tested in the M_DE/DS_ test, is linear at a constant drum rotation rate. They also demonstrated that an increase in the drum rotation rate causes an increase in the aggregate wear rate.

An analysis of the fragmentation process during the LA test was performed by Erichsen [16], who demonstrated its linearity. However, the experiment was performed for up to 900 rotations of the Los Angeles drum, and therefore more extensive research is recommended in order to understand the behavior of the rock material over a longer period of time.

Tugrul Tunc and Alyamac [72] estimated the fragmentation degree of mineral aggregates in the Los Angeles drum at different amounts of the grinding material (6, 12, 18, 24, 30 steel balls of uniform diameter) and at different rotation numbers of the test drum (500, 1000, 1500, and 2000 cycles). Based on the results, the authors concluded that in the case of the LA test, the number of steel balls and drum rotations has a significant influence on the value of the LA coefficient. The variability of the LA coefficient is so significant that together with an increase in the number of rotations and steel balls, the values of material loss due to abrasion increase up to 100%. The proposed preliminary estimation method allowed for predictions of abrasion-related loss of aggregate material at an expected level. This observation served as a basis to prepare an estimation that can be used to perform approximate, practical, and quick classifications of aggregates.

### 3.2. Influence of Crushing Processes on The Resistance to Wear and Fragmentation

Fragmentation and classification are essential processes allowing the production of mineral aggregates with a desired grain size (fraction). Having such a basic function, the above processes should also be analyzed from the perspective of their effectiveness. Aggregate production processes influence the geometric, physical, and mechanical properties of the produced material. At this point, a note should be made that issues related to the influence of the crushing processes (performed in crushers) on the strength parameters of rocks have not been yet extensively researched, as can be inferred from a limited number of relevant publications.

The properties of aggregates are largely determined by the geological origin of the deposit (its mineralogy, petrography) and only marginally by the production processes [73]. However, Miskovsky et al. [35] demonstrated that strength parameters of aggregates are influenced by microfractures and the content of minerals. Therefore, an improper production process can contribute to the destruction of the rock material and—as a result—to the lowering of its wear and fragmentation resistance. In addition, Rajan and Singh [36,37], Gawenda [32,74], Hofer et al. [33], Fernlund [31], and Räisänen and Mertamo [38] demonstrated that the type of the crushers and the variability of the crushing stages have an impact on the geometric parameters of mineral aggregates, significantly influencing the results of wear and fragmentation resistance.

Räisänen and Mertamo [38] observe that the shape parameters of aggregates from laboratory crushers may be inflated, leading to overestimations of aggregate quality. In conclusion, they suggest that the correlation between the laboratory and the industry-scale multi-step crushers cannot be identified. Thus, wear and fragmentation resistance may be different for the same rock material crushed under laboratory and industrial conditions.

Gawenda [32] indicates that the use of jaw crushers results in greater susceptibility to crushing, and aggregates are more easily crushed than with other crushing machines. Fladvad and Onnela [73] performed research into the influence of the parameters of a jaw crusher on the quality of crushed aggregates. They emphasize that geometric parameters can change with variable jaw crusher parameters. At the same time, they demonstrated that jaw crusher parameters have the least significant influence on the mechanical properties of aggregates. If a specimen is prepared in a laboratory crusher, however, its strength parameters are significantly affected. The quality of mineral aggregates can be optimized by adjusting the production process even to a single-step crushing process. Several crushing stages are needed to improve mechanical properties. Gawenda [32] emphasizes that in order to obtain better quality aggregates, multi-step systems should be pursued. The more crushing steps the raw material goes through, the more resistant it becomes to crushing in subsequent steps. This phenomenon is evidenced by the fact that as a result of the multi-step selective crushing process, aggregates are characterized by greater strength. However, this process also requires an increase in energy consumption in order to achieve the required grain size.

In his research, Köken [34] investigated the crushing of various types of rocks in order to determine how their resistance to fragmentation is affected. For this purpose, he identified particle size distributions for both the uncrushed and crushed material prior to and after the crushing procedure. Based on the obtained grain size distributions, he determined the degree of rock crushability and demonstrated that it increases together with the LA abrasion hardness.

### 3.3. Petrography and the Resistance to Wear and Fragmentation

The resistance to wear and fragmentation is largely influenced by the type of mineral aggregate used in tests and in practice. Generally, when selecting aggregates for different construction purposes, information on their geological and petrographic properties is one of the key aspects, as mechanical tests do not clearly describe any individual rock type [75]. Adomako et al. [6] extensively analyzed the relationships between the geology and the resistance to wear and fragmentation. Based on a literature review, they demonstrated that a large content of primary minerals (e.g., quartz and feldspar) in rocks should be considered an important parameter in evaluations of rock strength. Traces of secondary and accessory minerals also influence rock strength. Moreover, the influence of the mineral composition on the mechanical strength of rocks is insufficient to allow definitive conclusions on their mechanical properties, and therefore, other textural characteristics should also be considered. Analyses of the grain size distribution and the crystal size distribution of minerals (e.g., due to lithification) demonstrated that the resistance to wear and fragmentation increases in rocks having fine-grade textural structure ≤ 1 mm when compared to medium- and coarse-grained rocks (≥ 1 mm). Additionally, Nålsund [76] showed that the LA test cannot reveal the presence of weathered rock.

Hybrid rocks composed of a mixture of metamorphic and igneous lithotypes may be a significant reason behind physical and mechanical variability in the technological properties of aggregates. This variability may affect the performance and durability of structures constructed with the use of these aggregate rocks [77]. According to García-González et al. [2], the mechanical strength of aggregates is directly related to their mineralogy, porosity, and the metamorphosis degree of the source rock. In addition, Akseli and Leinonen [75] note that their tests of metavolcanic rocks suggest that the resistance to wear depends more on the mineralogical composition, while the resistance to fragmentation depends more on the changes of the texture.

Räisänen and Torppa [7], Sun et al. [15], Rigopoulos et al. [17], Akseli and Leinonen [75], Pang et al. [78], and Räisänen [79] observe a high correlation between aggregate properties and their mineralogical, petrographic, and textural properties. Sun et al. [15] conducted a pairwise correlation analysis and a multiple linear regression analysis in order to establish quantitative relationships between the mineral content and the morphological characteristics of aggregates. Rigopoulos et al. [17] proposed investigating the interrelations between the engineering parameters of construction aggregates using R-mode factor analysis. Their results suggest that factor analysis contributes to a deeper understanding of how the strength properties of aggregates change in-service. Akseli and Leinonen [75] observe that although the mineral composition is generally a key parameter, textural features, especially the mineral fabric and grain-size distribution, occasionally prove even more important. Research performed by Ajalloeian and Kamani [80] indicates that an increase of the Texture Coefficient (TC) is accompanied by a decrease in the Los Angeles Abrasive (LAA) coefficient. However, although a significant correlation is observed between the TC and the LAA, weak bivariate correlations exist between actual textural parameters and the LAA loss. Therefore, high values of textural parameters can be interpreted as a rock texture that influences the LAA loss.

Hofer et al. [33] emphasize that mineral composition has a significant influence on the results of fragmentation resistance tests. Additionally, Åkesson et al. [81,82] indicate that the size, shape, and spatial arrangement of grains are important parameters influencing the mechanical properties of rocks. Moreover, when defining the impact of mechanical parameters of rocks, consideration should be paid not only to the composition of rocks but also to their foliations.

Rock petrography has a significant influence on the variability of physical and mechanical parameters of aggregate depending on its fraction. This variability has been observed to be the lowest in aggregates obtained from homogenous and unweathered rocks. A higher degree of variability, on the other hand, is observed in aggregates obtained from rocks represented by different textural forms, here including rocks subjected to weathering processes [61].

### 3.4. Evaluation of the Quality and Properties of Aggregates with Respect to Wear and Fragmentation Resistance

Evaluation of aggregate quality is key to the evaluation of its usefulness. Prior to being used, each rock material must be tested for physical, mechanical, and geometric properties. These characteristics are defined by the composition and structure of the rock material, as well as by its exposure to the processes of physical and chemical weathering [17,79,81]. Undoubtedly, weathering processes in rocks affect the properties of aggregates. Alavi Nezhad Khalil Abad et al. [12] observe that good-quality aggregate should consist of particles having adequate strength and expected engineering properties and also be resistant to the exposure conditions. Importantly, some properties of aggregates show a high correlation between their own characteristics. Fournari and Ioannou [11] indicate that knowledge of several mineral aggregate characteristics can help predict, with high probability, the wear resistance of aggregate.

Mechanical strength should not be assumed constant for all products obtained from a particular deposit [73]. Differences observed in the analyzed aggregate properties, on the one hand, complicate their classification, but on the other hand, provide more detailed knowledge useful in evaluations of their application potentials and in the modeling of their wear processes. One of the methods for evaluating the quality of mineral aggregates is to use the Analytical Hierarchy Process (AHP) [9]. Alavi Nezhad Khalil Abad et al. [12] proposed the application of calculation techniques in evaluating the durability of limestone aggregates on the basis of their physical and mechanical properties. The method employs artificial neural networks and hybrid techniques of particle-swarm-optimization-based artificial neural networks. Rigopoulos et al. [17] indicate the potential for using factor analysis to investigate the relationship between aggregate properties, which is a method for evaluating mutual relationships between rock variables. It is a statistical method employed in descriptions of variability among observed variables in terms of a potentially lower number of unobserved variables called factors. For the purposes of identifying aggregate quality, Wu et al. [13] performed a virtual micro-Deval test with the use of the finite element method in the ABAQUS software. Based on this technique, a method was proposed for calculating virtual abrasion value, and the results of the simulations were consistent with the results of experimental tests. The simulation method is repeatable and operative and thus can be used in analyses of aggregate properties. On the basis of laboratory tests perfo rmed for aggregate from Alaska, Liu et al. [14] concluded that the implementation of the micro-Deval test in evaluations of rock durability provided satisfactory results, albeit they recommend it to be performed as an additional test at a certain period of time. From a long-time perspective, it should be used along with the LA test and with the sodium sulfate soundness test in order to ensure a more reliable evaluation of aggregate durability.

The quality of aggregate and the degradation process due to abrasive action in the LA drum can be visualized by plotting the results of this degradation in a triangular diagram. Apart from recording the Los Angeles value (LA < 1.6 mm), measurements can also be taken of the material remaining in its original test fraction (the Los Angeles remaining mass, R_LA_, material > 10 mm), and calculations can include the amount of the material in the intermediate fraction (the Los Angeles intermediate, I_LA_, material 1.6–10 mm). The three values (LA, R_LA_, and I_LA_) add to reach 100%, and they can be represented in a triangular diagram. With a view to practical applications of aggregates, it seems reasonable to focus on the amount of the material remaining in the original range of the R_LA_ test fraction [16]. Identifying this amount allows a more accurate representation of the behavior of aggregate and its susceptibility to crushing.

Evaluations of the parameters describing the wear and fragmentation resistance of dry and saturated aggregates seem justified, as the material is later used in various environmental conditions. In this context, Palassi and Danesh [8] demonstrated that saturated aggregates are more prone to fragmentation. They trace the reason for this phenomenon to the fact that friction between aggregate particles decreases when aggregate is saturated. A similar comparison was performed for the M_DE/DS_ test by Strzałkowski [83] and Woodside and Woodward [84]. They demonstrated that the abrasion value of saturated aggregates is greater than the abrasion value of dry aggregates.

García-González et al. [2] provided geomechanical characteristics of aggregates of igneous origin and compared them with other properties of these materials. Their work demonstrated that an increase in their porosity causes a change in the mechanical behavior of aggregates. The influence of high porosity on faster fragmentation in the LA test was also confirmed by Adomako et al. [6], Kahraman and Fener [59], and Khaleghi Esfahani et al. [85]. In addition, Czinder and Török [3] and Ugur et al. [52] stress that the mechanical parameters of rock materials show a high correlation with their bulk densities.

Czinder and Török [3], Capik and Yilmaz [41], Hydzik-Wiśniewska and Bednarek [46], Török and Czinder [50], Tuncay et al. [51], Ugur et al. [52], and Török [86] conducted their research on aggregates from various sources. Based on the obtained results, they demonstrated a strong correlation between the resistance to fragmentation and the resistance to wear, as well as between the resistance to fragmentation or wear and the resistance to compression. A noticeable correlation can also be observed between the resistance to fragmentation on the one hand and the aggregate crushing value and aggregate impact value on the other hand [8,87].

The geometric dimensions of aggregates are also important for their strength parameters [88]. In general, geometric indicators of aggregates (the shape and flatness indexes) are highly correlated with their strength indexes. The higher the content of flat and longitudinal grains, the greater the abrasion value (the lower the resistance to fragmentation). This hypothesis was confirmed by Bulevičius et al. [40]. Qian et al. [67], Zhang et al. [89], Deiros Quintanilla et al. [90], Guo et al. [91], Ge et al. [92], Wang et al. [93,94], Boler et al. [95], Lane et al. [96], and Tolppanen et al. [97] analyzed the wear and fragmentation processes performed with the use of the LA and micro-Deval testing machines. Some of the above researchers used 3D techniques. They observed a change in the size and surface roughness of aggregate grains. Their publications indicate that during the first phase of the wear and fragmentation processes, the aggregate grains have sharp edges and ends, which are smoothened and reduced in volume as the test progresses. Wang et al. [94] stress that a log-normal function is ideally suited to describe the analyzed morphological characteristics before and after the identification of the micro-Deval coefficient. The change of angularity is the main reason behind the loss of mass, while the changes of both the sphericity and texture only have an additional influence. Cavalcanti et al. [98] indicate that the sphericity (cubicity) of aggregate grains after the M_DE/DS_ test does not change significantly, and this fact may be due to the smaller size of the abrasive material. Additionally, the surface texture after the abrasion test in the LA drum is not significantly different than in the case of the grains prior to the test, and this fact may be accounted for by their tendency to crack during the test.

Abdelhedi et al. [99] demonstrated a non-destructive ultrasound technique of identifying the mechanical strength of carbonate aggregates, which is mainly measured in the LA tests and in the micro-Deval tests (the M_DE/DS_ test). The ultrasound tests, performed with the use of longitudinal wave P, were linearly correlated with the LA and M_DE_ coefficients. The application of ultrasound techniques allows predictions of the mechanical properties of aggregates except for long-term tests of resistance to wear and fragmentation. This solution seems to allow effective evaluations of aggregate quality at each stage of their use.

The relationship between polishability and the resistance to wear and fragmentation is at a high correlation level [44,45,100]. These relationships enable an evaluation of the mechanical parameters of aggregates with the use of a coordinate system and with allowance for the threshold values of polishability and abrasion. As a result, it is possible to identify aggregates of both low and high mechanical strength, i.e., those of high and low resistance to wear and fragmentation [68].

Laboratory tests indicate that simulated freezing–thawing cycles significantly affect the properties of rocks. The tests demonstrate that such parameters as the influence of magnesium sulfate or sodium sulfate, as well as the LA tests, account well for the mechanism of aggregate fragmentation [14,39,48]. A similar influence of magnesium sulfate on wear in micro-Deval tests was confirmed by Fournari and Ioannou [11], Durmeková et al. [43], and Wu et al. [101]. Czinder and Török [42] observe that the influence of magnesium sulfate may cause a loss of abrasion hardness of as much as 35%.

The literature also indicates that a correlation between the resistance to wear and fragmentation on the one hand and other aggregate parameters on the other hand is not always visible or is only weak [43,102,103]. This fact may be a result of different test methods or different rock types, which have various structures and compositions. Therefore, when evaluating the quality-related parameters of aggregates, special attention should be paid to the geology and petrography of the tested material.

## 4. Discussion and Conclusions

Aggregate wear and fragmentation tests are performed according to different standards in force worldwide. The standards followed in Europe are EN 1097-1 [53] and EN 1097-2 [54], but other countries follow such standards as: ASTM D6928 [55], AASHTO T327 [56], ASTM C131/C131M [57], and AASHTO T-96 [58]. Such a situation renders a comparison between results obtained with the use of different test methods impossible, as these results depend on the material and the grain size of the investigated aggregates.

Tests of resistance to wear and fragmentation are performed in the micro-Deval apparatus (the M_DE/DS_ test) and in the Los Angeles drum (the LA test). Comparative tests demonstrated that the M_DE/DS_ test is more effective than the LA test, as the test conditions are more realistic. The disadvantage of both tests lies in the limited dimension range of the aggregate. It is a significant problem, as aggregates show different mechanical properties depending on their fraction. This fact leads to uncertainty as to whether a material of greater grain size is subjected to identical degradation as a material of smaller grain size. The use of steel balls in the LA tests can also be questioned. Their application may not reflect the influence of the internal properties of the material on its fragmentation characteristics.

Test duration is also an object of disagreement. In the case of the M_DE/DS_ test, the duration is short, and as a result, the tested aggregate shows linear abrasion hardness. However, the long-term tests reveal that this phenomenon has an exponential character. Importantly, the longer the test duration (the more abrasion cycles), the smaller (gradually) the effect of aggregate-to-aggregate wear. A similar situation is observed in the case of the LA test, whose short-term (up to 900 rotations) results also indicate a linear fragmentation process. In contrast, preliminary tests performed for various numbers of drum rotations (up to 2000) reveal that an increased number of rotations and steel balls may cause the value of the LA index to grow by as much as 100%.

Apparently, key factors influencing the wear and fragmentation of aggregates include the geological properties of the deposit (its mineralogical composition, as well as the size, shape, and arrangement of grains). Microfractures also deserve special attention, as they may cause a decrease in the quality of the aggregate during the production process. The quality of the aggregate is also influenced by the type of the crushers. The quality of aggregate from laboratory crushers is too high in comparison to the quality of industrial aggregates, making their comparison impossible. However, the use of jaw crushers and of a single-step crushing process may increase the quality-related parameters of the produced aggregate.

The qualitative evaluation of mineral aggregates is a complex issue, as it should be based on the analysis of various aggregate properties. These should be additionally compared between various types of aggregates that have various geological characteristics. The geomechanical properties of rocks (wear and fragmentation resistance) are insufficient to indicate potential applications of aggregates beyond any doubt and to predict their lifetime. Although the geomechanical properties of rocks are correlated with other physical, mechanical, and geometric characteristics, a mathematical relationship common to all aggregates cannot be defined explicitly.

Broad analyses presented in this publication describe methods for identifying the resistance of aggregates to wear and fragmentation and the behavior of rock material over the duration time of the tests and suggest further research steps to be undertaken in order to develop improved test procedures reflecting the conditions in which aggregates are practically applied:

- Proposals of changes that would improve and systematize the test methods, involving broadly defined studies on an optimal research methodology;
- A broad analysis and a selection of an optimal test method that would allow for a broad dimension range of aggregates tested for wear and fragmentation;
- Analysis of short-term and long-term wear and fragmentation tests in relation to changes in the values of the coefficient describing the resistance to wear and fragmentation on a large population of rock materials;
- Extensive research into the impact of the production process (crushing in crushers) of aggregates on their physical and mechanical properties;
- A broad analysis of physical, mechanical, and geometric parameters of aggregates together with their interrelationships, with particular focus on the geological properties and an attempt at determining mathematical relationships, which may help to perform a qualitative analysis of granular rock materials.

## Figures and Tables

**Table 1:** Keywords and search results.

Years	Keywords		
	“Los Angeles AND Aggregate AND Resistance to Fragmentation”	“Micro-Deval AND Aggregate AND Resistance to Wear”	“Aggregate AND Micro-Deval AND Los Angeles”
	Number of Documents		
1990–1994	1	1	1
1995–1999	0	3	3
2000–2004	6	4	6
2005–2009	7	7	16
2010–2014	17	15	36
2015–2019	27	57	66
2020–2021 ^1^	8	23	34
Total	66	110	162

^1^ Analysis performed on 1 February 2021.

**Table 2:** Comparison of test parameters (own study based on Gökalp and Uz [4], Gökalp et al. [29], Wu et al. [30]).

Micro-Deval Test		
Parameters	EN 1097-1	ASTM D6928
Aggregate size distribution	For A grading: 4.0–6.3 mm For B grading: 4.0–8.0 mm For C grading: 6.3–10.0 mm For D grading: 8.0–11.2 mm For E grading: 10.0–14.0 mm ^1^ For F grading: 11.2–16.0 mm For G grading: 31.5–50.0 mm ^2^	For A grading: 19.0–9.5 mm For B grading: 12.5–4.75 mm For C grading: 9.5–4.75 mm
Aggregate mass	For A-F grading: 500 ± 2 g For G grading: 10 000 ± 100 g	1500 ± 5 g
Sieve size for final evaluation	1.6 mm	1.18 mm
Mass of ball load	For A grading: 2000 ± 5 g For B grading: 2800 ± 5 g For C grading: 4000 ± 5 g For D grading: 4400 ± 5 g For E grading: 5000 ± 5 g For F grading: 5400 ± 5 g For G grading: without ball load	5000 ± 5 g
Amount of water	For A–F grading: 2.5 ± 0.05 l For G grading: 2.0 ± 0.05 l	2.0 ± 0.05 l
Revolution numbers	For A–F grading: 12 000 ± 10 For G grading: 14 000 ± 10	For A grading: 12 000 ± 100 g For B grading: 10 500 ± 100 g For C grading: 9500 ± 100 g
Calculation of M_DE/DS_ coefficient	For G grading: $M_{DE/DS}=\frac{500-m_{1}}{5}$ For G grading: $M_{DE/DS}=\frac{10000-m_{1}}{100}$	$M_{DE/DS}=\frac{1500-m_{2}}{1500}\cdot 100$
**Los Angeles Test**		
**Parameters**	**EN 1097-2**	**ASTM C131**
Aggregate size distribution	For A grading: 4.0–6.3 mm For B grading: 4.0–8.0 mm For C grading: 6.3–10.0 mm For D grading: 8.0–11.2 mm For E grading: 10.0–14.0 mm ^1^ For F grading: 11.2–16.0 mm For G grading: 31.5–50.0 mm ^2^	For A grading: 37.5–9.5 mm For B grading: 19.0–9.5 mm For C grading: 9.5–4.75 mm For D grading: 4.75–2.36 mm
Aggregate mass	For A–F grading: 5000 ± 5 g For G grading: 10 000 ± 100 g	5000 ± 10 g
Sieve size for final evaluation	1.6 mm	1.7 mm
Mass of ball load	For A grading: 2930–3100 g For B grading: 3410–3540 g For C grading: 3840–3980 g For D grading: 4250–4420 g For E grading: 4690–4860 g For F grading: 5120–5300 g For G grading: 5120–5300 g	For A grading: 5000 ± 25 g For B grading: 4584 ± 25 g For C grading: 3330 ± 20 g For D grading: 2500 ± 15 g
Revolution numbers	For A–F grading: 500 ± 10 For G grading: 1000 ± 10	500 ± 10
Calculation of LA coefficient	For A–F grading: $LA=\frac{5000-m_{1}}{50}$ For G grading: $LA=\frac{10000-m_{1}}{100}$	$LA=\frac{5000-m_{3}}{5000}\cdot 100$

^1^ Basic aggregate fraction, ^2^ tests of railroad ballast aggregates, m_1_—mass of dry aggregate remaining on the 1.6 mm sieve, m_2_—mass of dry aggregate remaining on the 1.18 mm sieve, m_3_—mass of dry aggregate remaining on the 1.7 mm sieve.

## Data Availability

No new data were created or analyzed in this study. Data sharing is not applicable to this article.
